# Sleep Action Recognition Based on Segmentation Strategy

**DOI:** 10.3390/jimaging9030060

**Published:** 2023-03-07

**Authors:** Xiang Zhou, Yue Cui, Gang Xu, Hongliang Chen, Jing Zeng, Yutong Li, Jiangjian Xiao

**Affiliations:** 1 Faculty of Electrical Engineering and Computer Science, Ningbo University, Ningbo 315211, China; 2 Computer Vision Laboratory, Advanced Manufacturing Institute, Ningbo Institute of Materials Technology and Engineering, Chinese Academy of Sciences, Ningbo 315211, China

**Keywords:** sleeping behavior recognition, long-term memory network, self-attention coding, segment-level feature fusion

## Abstract

In order to solve the problem of long video dependence and the difficulty of fine-grained feature extraction in the video behavior recognition of personnel sleeping at a security-monitored scene, this paper proposes a time-series convolution-network-based sleeping behavior recognition algorithm suitable for monitoring data. ResNet50 is selected as the backbone network, and the self-attention coding layer is used to extract rich contextual semantic information; then, a segment-level feature fusion module is constructed to enhance the effective transmission of important information in the segment feature sequence on the network, and the long-term memory network is used to model the entire video in the time dimension to improve behavior detection ability. This paper constructs a data set of sleeping behavior under security monitoring, and the two behaviors contain about 2800 single-person target videos. The experimental results show that the detection accuracy of the network model in this paper is significantly improved on the sleeping post data set, up to 6.69% higher than the benchmark network. Compared with other network models, the performance of the algorithm in this paper has improved to different degrees and has good application value.

## 1. Introduction

In recent years, with the rapid growth of video surveillance data and the rapid development of artificial intelligence, big data, the Internet of Things and other technologies, society’s emphasis on public safety has gradually deepened. Intelligent perception technology based on video surveillance has become important. Intelligent sensing technology based on video surveillance has become an important direction of the development of security monitoring [1], and is widely used in smart cities, smart campuses, the smart chemical industry and other fields and industries [2,3,4,5,6,7,8,9,10,11,12,13]. In the face of massive video surveillance data, the personalized security task requirements of different scenes, and the algorithm performance requirements of industrial applications, traditional security technology has been unable to meet the current market demand. Therefore, with the vigorous development of intelligent technology and parallel computing, as well as the continuous updating of monitoring equipment, intelligent security technology based on deep learning has emerged at this historic moment. Compared with traditional security technology, vision algorithms based on deep learning have the significant advantages of higher network recognition accuracy and efficiency and stronger model generalization ability. At the same time, the field of intelligent security also shows great scientific research value and broad industrial application prospects.

Human target detection and behavior recognition are the most common basic problems in security tasks. Because of the subjective initiative and non-rigid characteristics of humans, this is a complex research topic in the field of computer vision. Human detection detects and locates a human body from video, and behavior recognition identifies and analyzes human behavior. Human body detection is a basic component of intelligent processing systems, such as security and transportation. The real-time and accurate detection of human body targets is also the basis for realizing human abnormal behavior analysis and other visual tasks. Behavior recognition is an important part of personnel intelligent management systems. The automatic and real-time intelligent monitoring of personnel using video can greatly improve personnel management levels. In practical work, sleeping on duty can potentially lead to safety hazards, especially when sleeping on duty at night causes safety accidents. By using a sleeping behavior recognition algorithm, the sleeping condition of the personnel on duty can be detected, and corresponding preventive measures can be taken to reduce the occurrence of accidents. The adoption of a sleeping behavior recognition algorithm can digitally display the working state of on-duty personnel, improve supervision efficiency, and avoid dangerous behaviors caused by the subjective factors of the on-duty personnel not being detected and stopped in time, which will lead to serious consequences.

In this paper, we propose a long video classification network incorporating a self-attention mechanism. This method is divided into two stages, feature extraction and feature fusion enhancement, which can effectively realize the transmission of important information in the segment feature sequence. In the feature extraction stage of a CNN (convolutional neural network), we first added the self-attention mechanism module to obtain the fine-grained features between long video sequences, solving the problems caused by difficulty in extracting the fine-grained features of people’s sleeping behaviors. In the feature fusion stage, the segmentation strategy module was added to effectively solve the problem of long-distance dependence of spatiotemporal information in long video feature fusion. The experimental results show that this algorithm improves the transmission ability of the network segment feature sequence, further improves the CNN-LSTM (Integration of convolutional neural network and long-term memory network) network’s detection accuracy of sleeping behavior, and has high practical application value.

The contributions of this work are summarized as follows:

- An overview of methods, models and algorithms used in personnel behavior recognition based on deep learning;
- A sleeping post data set built under the monitoring situation for the training of a sleeping personnel post task network model;
- For the task of recognizing sleeping personnel behavior, a sleeping behavior recognition algorithm based on a self-attention time convolution network for monitoring data is proposed: 1. In the feature extraction stage of a CNN network, the self-attention mechanism module is added to obtain the fine-grained features of the image, so that the feature extraction network pays more attention to the movement features around the people, solving the problems caused by difficulty in extracting the fine-grained features of people’s sleeping behaviors. 2. In the feature fusion stage, a video segmentation strategy is proposed, which uses segment-level features to stack into video-level features to effectively solve the problem of long-distance dependence of spatiotemporal information in long video feature fusion.

The rest of this paper is organized as follows: Section 2 describes the methods of human behavior recognition based on monocular cameras, as well as various previous methods of sleep detection. Section 3 describes the introduction of the innovative network model module in this paper. Section 4 describes the evaluation of our proposed network model and other networks based on the sleeping post data set constructed in this paper, and gives the experimental results. Section 5 summarizes this paper.

## 2. Related Work

The feature extraction of the early traditional image processing method [14] mainly relies on artificially designed feature extractors. It is for specific applications in specific scenarios. The generalization ability and robustness of the model are poor. Compared with traditional image processing methods, abnormal testing research based on deep learning has attracted much attention. This algorithm can automatically learn various fusion features from a large number of training samples by designing appropriate deep neural network models. It is relatively environmentally robust and can overcome some disadvantages of traditional methods. Ji et al. [15] proposed the concept of 3D convolution and used it for behavioral identification. Based on fully convolutional neural networks (FCNS), a fully convolutional network obtained using full supervision pre-training of another unsupervised full convolutional network was transferred to obtain a new convolutional network to detect abnormal events on video. Additionally, Sabokrou et al. [13] proposed the unsupervised method of using an automatic encoder (AUTO-ENCODERS, AES) to learn normal behavior and used sample reconstruction to determine the unsupervised method of unusual behavior. Luo et al. [16] used the long short-term memory (LSTM) model to capture temporal features, memorize the action information of the time dimension, and achieve more accurate abnormal behavior pattern learning. However, when dealing with long time series alone, it is still possible to lose important features. Jeff et al. [17] used LSTM for two streams, but the final result was still obtained through later fusion. According to the framework of CNN-LSTM, the following researchers proposed several variants, such as bidirectional LSTM [18], CNN-LSTM fusion [19] and a layered multi-granularity LSTM network [20]. Video LSTM [21] includes a spatial attention mechanism based on a correlation and attention mechanism, in turn based on lightweight motion. Ma et al. [22] established a strong baseline for fair comparison and deeply studied the effect of learning spatiotemporal characteristics, and found that the depth of the LSTM network needs to be appropriately increased to improve network performance. The dual-flow algorithm proposed by Simonyan [23] creatively uses different methods to process the time–space dimension information. The spatial dimension input uses a single-frame RGB to process the information, and the temporal dimension input uses a multi-frame density optical flow field as the input CNN to process the information. The dense optical flow is used to represent the action characteristics, and the data set is combined through the multi-task training method to remove over-fitting and obtain better results. Wang et al. [24] found that the dual-stream network can only deal with short-term movement, the information understanding in the long-term movement is not enough, and the training samples included were not complete. Therefore, the sparse time sampling strategy and the strategy based on video surveillance were adopted to randomly extract fragments from the video source stream after time domain segmentation to make up for the first deficiency, and the cross pre-training, regularization technology and data expansion technology were used to make up for the second deficiency. This network structure was referred to as temporary segment networks (TSN). Feichtenhofer et al. [25] proposed STRESNET, which deals with the pixel-level correspondence of the time domain and spatial domain. First, it was extended in the time domain, and continued the characteristics of the fusion network, resulting in relatively excellent results. Then, the authors of [26] also proposed the SLOWFAST network, which uses low-frame-slow and high-frame-fast approaches to extract spatiotemporal features, and achieved relatively ideal results. The SLOWONLY [26] network, which only uses slow branches, also achieved good performance.

Although a great deal of research has been carried out in the field of video behavior recognition, there are still great challenges in the recognition of drowsy abnormal behavior in the monitoring environment. The difference between sleeping behavior and general behavior is that sleeping behavior lasts for a long time, sleepers produce less motion information within a certain period of time, and that sleeping behavior is highly similar to the representational information and spatiotemporal characteristics of daily office behavior. Additionally, because the monitoring devices are often far away, it can be difficult to clearly observe the state of the face, which makes it difficult to recognize sleeping behavior through face judgment.

For sleep scenarios, most sleep detection studies [27,28,29] have used EEG (electroencephalogram) and various professional sleep data as network inputs to identify and analyze sleep behavior and sleep status. This is difficult to deploy and implement in real life, so some studies based on computer vision have been produced. Most of these studies were realized using traditional image processing. For example [30], proposed using the three-frame difference method to detect and analyze students’ sleeping behavior in class, and [31] used a single frame of an image as the input of the detection network, took the sleeper as the label of the detection network, and then counted the number of frames containing the sleeper image. When the number of frames reached a certain threshold, the sleep behavior was determined. This method uses sleep behavior as the detection of a single image in a video sequence, which is greatly affected by the performance of the detection network. The disadvantage of this method is that the posture and angle of view are different during sleep, and the detection network pays more attention to the representational information on the image and completely ignores the timing information in the video, resulting in an unsatisfactory network effect. We regard sleeping behavior as a kind of behavior in which people remain in the same state for a long time. However, it is difficult to extract fine-grained motion features in long video, and there is an additional problem of long-distance dependence, which leads to the poor performance of the entire network. To solve these problems, we propose a segmentation feature fusion strategy, and because of the high similarity between the single-frame images of sleep behavior, the similar features are retained when calculating the fragment-level features. Additionally, sleep is a long and relatively static action, so it is very difficult to sample video intensively, and it consumes a lot of computing power.

## 3. Method

### 3.1. Local Feature under Self-Attention Mechanism

**Figure 1:**
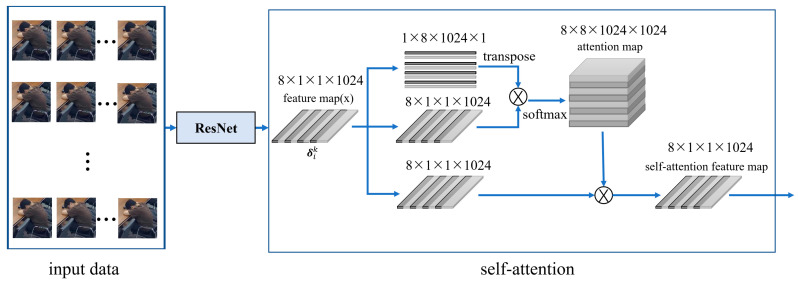
Self-attention mechanism module.

People’s understanding of motion video comprehensively describes their motion information. Understanding the feature association between video frames is essential to improve the accuracy of video motion recognition. In a time series, not all information is equally effective. The previous information can be used to help understand the subsequent content. LSTM is widely used in the research of human behavior recognition because it can process long time series data and has good memory ability. Therefore, we chose LSTM as our final decoding module. The excellent structure of LSTM can ensure that the sequence path from the previous unit to the current unit still exists, meaning that the network can provide a large amount of long-term information compared with other RNN networks. However, in fact, LSTM can only remember limited distance information at most. For longer-distance information, LSTM has a general effect and is obviously insufficient in image feature extraction. As a special case of an attention mechanism [32], self-attention can learn its own representation through only a single sequence in a deep-learning-based natural language processing task [33], from which we can obtain inspiration. In the network coding stage, the self-attention mechanism is introduced after the data pass ResNet50 to avoid the long-distance dependence problem caused by a long image sequence. Its network structure is shown in Figure 1.

The self-attention score was calculated and aggregated according to Equations (1)–(3) to obtain the output result of the self-attention mechanism represented by a feature vector. Assuming that the input feature is $\delta_{i}^{k}$, the self-attention score was calculated based on the following formula:

(1) $$\gamma =(g{\left(\delta_{i}^{k}\right)}^{T}\cdot y(\delta_{i}^{k}))$$

(2) $$score=softmax(\gamma /\sqrt{D})$$

(3) $$AT\left(u_{i}\right)=score\times conv\left(\delta_{i}^{k}\right)$$

Among them: $\delta_{i}^{k}$ represents the feature vector of the image frame sequence through the output of the Resnet encoding module 8 × 1 × 1 × 1024; *g*(*·*) and *y*(*·*) are linear transformation operations (here is a convolution operation of 1 × 1); γ represents the similarity between the feature of $\delta_{i}^{k}$ after convolution; *D* stands for the number of output channels, except for the regulation of *D*, so that the training process has a more stable gradient; score is a self-attention score of $\delta_{i}^{k}$, which is calculated using the normalized index function *softmax*; and *conv*(*·*) means 1 × 1 convolution. Finally, we weighed the weight and the corresponding value and obtained the self-attention score of 8 ×1 × 1 × 1024.

### 3.2. Fragment-Level Feature Fusion Module

In video classification, the length of the video sequence will significantly impact the network performance. A convolutional network is powerless to model a long-range time structure, mainly because it uses only a single-frame image stack or only one frame in a short segment. Hence, access to the space–time context is limited. Although a temporal segment network (TSN) [24] effectively improves the network’s ability to extract global features by segmenting the video, the TSN [24] still includes all information in the clip in the final fusion stage, including some redundant features, and uses a random sampling method to sample image frames in the clip, resulting in a loss of video-level features to a certain extent. The long-distance dependence of long video has not been effectively solved. The advantages of segmented networks have not been fully utilized.

**Figure 2:**
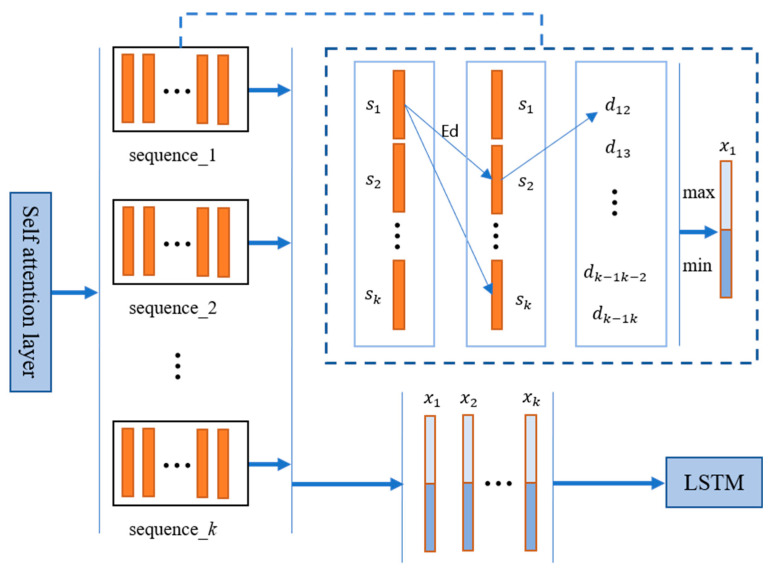
Clip-level features stacked into video-level features. $x_{1}$–$\;x_{k}$ are the segment-level features of sequence 1 − *k* output through segment module; $s_{1}\;$–$\;\;s_{k}$ are frame-level features of the image in each segment.

Aiming at the problems in TSN [24], we propose a method based on feature similarity to select frame-level features to replace the original random sampling method in clips. After the CNN network, the Euclidean distance between feature vectors was used to represent the similarity between features. For each clip, we selected the most representative feature (that is, the one with the smallest Euclidean distance from other frames of the current clip) and the most dissimilar feature (the one with the largest Euclidean distance from other frames of the current clip) as the two key features of the current clip, connecting the two features as the clip level feature representation of the current clip. We believe that a video clip contains both features with large changes and features with small changes. Similar features in the video sequence usually represent the static features of the person being focused on, who will show weak movement during sleep. If only the features with large changes are considered, the similarity between the two kinds of dormant and non-dormant behaviors will be increased, resulting in poor network performance. The frame-level feature filtering method is shown in Figure 2.

When the segment-level feature fusion module divided the video sequence into multiple segments, we used 4 frames, 8 frames and 16 frames as the segment length of the entire video sequence. If the segment length is too short, the similar feature vectors in the entire sequence will still have redundancy, resulting in the advantages of the segmentation strategy not being fully reflected; however, a segment that is too long will not only lead to an exponential increase in the amount of computation, it will also cause the loss of some important features. After our experiments, we finally chose 8 frames as the video sequence partition interval. Although we increased a part of the computation amount during segment division, we selected most features before entering LSTM, which reduced the number of feature vectors entering the LSTM layer, so as to not reduce the efficiency of the overall network.

**Figure 3:**
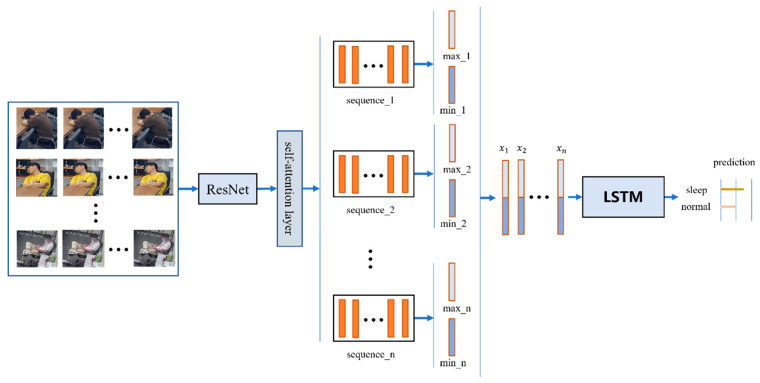
Overall structure of video classification network in this paper; $x_{1}-x_{n}$ represents fragment-level features (LSTM: long short-term memory).

The picture frame is output as a feature vector of 1 × 1024 through the CNN encoding layer. We divided a feature sequence of the completed video into equal-length segment sequences and then calculated the distance between all feature vectors and other feature vectors in each segment:

(4) $$d_{ij}=S_{i}•S_{j}=\overset{n}{\underset{k=1}{\sum }}a_{k}b_{k}$$

where $S_{i}=\left(a_{1},a_{2},\ldots ,a_{n}\right);\;S_{j}=\left(b_{1},b_{2},\ldots ,b_{n}\right)$, and we obtained the distance between all feature vectors and other feature vectors $Dis=(d_{12},d_{13},\ldots \;,d_{1n},d_{23},d_{24},\ldots ,d_{2n},\ldots \;,d_{n-1n})$. We calculated the total distance between the current vector and other vectors according to Equation (5) to form the distance queue SumDis, and took the feature vector corresponding to the maximum and minimum value of SumDis to connect into a 1 × 2048 feature vector as the final segment-level feature to be stacked. The process of stacking clip-level features into video-level features is shown in the dotted box in Figure 3.

(5) $$SumDis=\left(D_{1},D_{2},\;\ldots \;,D_{n}\right)=(\overset{n}{\underset{k=1}{\sum }}d_{1k},d_{12}+\overset{n}{\underset{k=1}{\sum }}d_{2k},\ldots ,\overset{n}{\underset{k=1}{\sum }}d_{kn})$$

### 3.3. Network

The video classification network model proposed in this paper is based on CNN-LSTM and consists of two modules: a CNN-based encoder and an LSTM-based decoder. The overall structure design of the network is shown in Figure 3. In the CNN coding stage, ResNet50 is used as the backbone network for feature extraction. A self-attention mechanism is added after Resnet50 to solve the network long-distance dependency problem to obtain better feature representation of single-frame images in clips. Then, the segmentation strategy and feature similarity method are used to filter the frame features of each segment to obtain segment-level features, which can improve the learning ability of the network model for local key features. Then, the segment-level features are stacked to form video-level features to enhance the network’s capture of the overall structural features. Finally, the video-level features are input into the LSTM network to decode and construct the conversion relationship between the video-level features and the marking results.

## 4. Experiment

### 4.1. Experimental Platform

This paper implemented the computing environment configuration as follows: hardware (CPU: Intel Core i9-10920X, GPU: GTX 2080Ti), software (python 3.7, deep learning framework pythoch 1.9, opencv 3.4, ffmpeg), and software tools (Visual studio 2017, PyCharm 2020). The optimizer used for network training was the Adam optimizer. The initial learning rate was 1 × 10^−4^. A total of 100 epochs were iterated. The early stop method was used to avoid overfitting.

### 4.2. Data Set Creation

**Figure 4:**
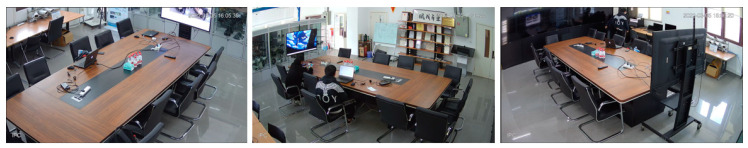
Image collection effect of laboratory monitoring camera.

In this paper, a long video action behavior recognition algorithm based on the video segmentation strategy was studied, mainly focusing on detecting abnormal sleepiness in video surveillance. For this study, the current public data set could not meet the requirements well. Due to the existence of the long tail effect, although the public data set also contains common abnormal behaviors, the amount of data was very small, and it could not be well-trained for the abnormal sleepy behaviors covered in this paper. Although the method in this paper was to identify the sleeping behavior of the monitored individuals from a single perspective, considering the discreteness of the data set and the number of data samples, we used monitoring videos from different perspectives to build the data set. To this end, three surveillance cameras were installed in three corners of the laboratory to build a data acquisition environment to obtain video of sleepy behavior, and a video data set was built. Figure 4 is an example of an image obtained by a surveillance camera in an experimental environment.

**Figure 5:**
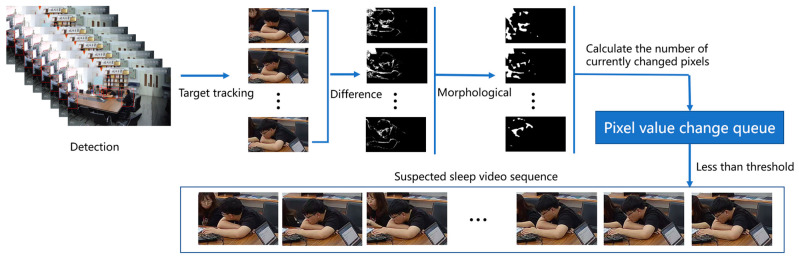
Video data set construction process.

The environment under video surveillance is more complex, with many personnel targets. As an independent individual, each person has different characteristics. If the original image is used as the training set, it will inevitably cause a lot of feature redundancy and reduce the network recognition ability. Therefore, in establishing a data set, capturing the suspected sleepy people from the video for initial screening is necessary. In this paper, the following methods were used to capture specific targets. The data set establishment process included six steps, as shown in Figure 5.

The description of sleeping behavior under the surveillance video is usually a single person’s behavior state with small action amplitude or relatively static behavior for a long time. When performing sleep recognition analysis, we should pay more attention to the changes in a single person’s front and back frames. Firstly, the YOLOv4 target detection network was used to detect and track the original video image, and the single-person target frame was extracted to form a video image sequence; considering the high cost of optical flow calculation, the inter-frame difference method was used to obtain the contour of the moving object from two consecutive frames of the video image sequence. When the object moved in a large range, there were obvious differences between the two adjacent frames. The expansion and erosion algorithm was used for morphological processing to enhance and eliminate noise. The absolute value of the pixel value difference was obtained at the corresponding position of the image sequence; the median value and average value of the pixel were calculated; it was judged whether they were less than a certain threshold; and then the image sequence that belonged to the suspected sleeper was extracted. In reality, due to the long distance between the surveillance cameras, people who have not worked for a long time will also be misjudged as likely to sleep, so it was necessary to manually filter the data into two categories, sleeping behavior and normal behavior, to complete the construction of the video data set.

Depth learning methods usually improve the accuracy with the increase in training data. For video action recognition, this means that large-scale labeled data sets are needed to train effective models. Due to the difficulty of self-collecting data, the number of sleepy video data sets under monitoring was small, and the self-collected sample data only contained 306 video clips. Therefore, this paper considered the method of changing the frame and taking the time of video clips to expand the data set to increase the sequence of sleepy images. In this paper, video images were taken at intervals of 5 frames, 30 frames and 3 different forms of video samples of their mixed frame combinations as data sets, and sent to the CNN-LSTM network for training. The experimental results are shown in Table 1.

**Figure 6:**
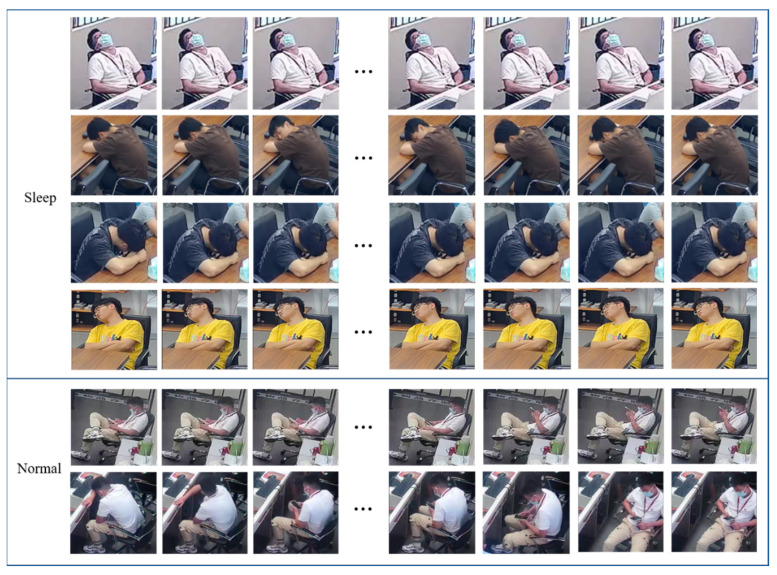
Schematic diagram of image sequence of sleeping behavior and daily behavior.

It can be seen from Table 1 that the classification accuracy of CNN-LSTM reached 89.20% on the data set with 5 frame intervals, and the accuracy of comparison between 30 frames and hybrid mode improved by 3.38% and 3.75%. It can be considered that a large number of similar but different samples could be obtained by controlling the interval between frames, and more video samples with better effects could be obtained by adding irregular noise. Based on this method, the data set was expanded to 2800 groups of video clips, each of which had 1800–5400 frames. The image sequence data set of some sleeping behaviors and daily behaviors is shown in Figure 6.

### 4.3. Description of Evaluation Indicators

The behavior recognition network model accuracy evaluation index uses the accuracy rate to evaluate the video classification network. The calculation formula is as follows:

(6) $$Accuracy=\frac{TP+TN}{TP+TN+FP+FN}$$

where: *TP* represents the number of real categories that are actually real and predicted; *TN* refers to the number of error categories predicted to be error categories; *FP* represents the number of actual error categories that are predicted to be real categories; and *FN* indicates the number of correct categories that are predicted to be wrong categories.

### 4.4. Results and Analysis

In the sleep data set, all video samples contained only a single target. The sleep state and other states were selected as the classification data set. The two behaviors included about 2800 videos, of which 1988 videos were used as the training set, and 426 videos were used as the test set. The distribution of experimental data is shown in Table 2.

**Figure 7:**
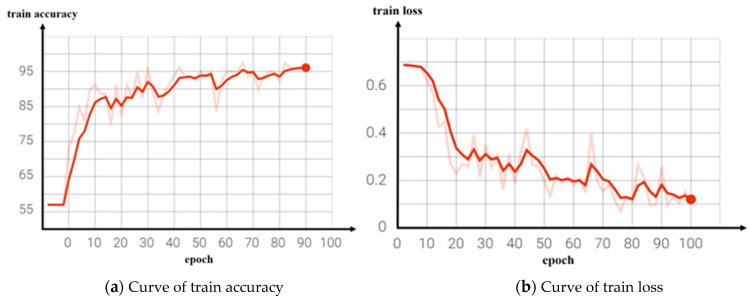
Curve of benchmark network training results.

**Figure 8:**
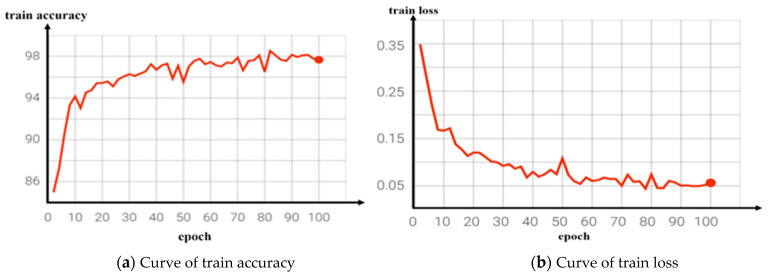
Curve of improved network training results.

We conducted training models for both the reference network and the improved video classification network and output the corresponding classification accuracy curve and loss decline curve during the training process, as shown in Figure 7 and Figure 8. Figure 7b is the loss decline curve of the benchmark network training. It can be seen that the convergence effect of the loss value was poor and the convergence speed was slow after 100 network iterations, while Figure 8b is the training loss curve of the improved network. It can be seen that the loss value of the model tended to converge with the increase in the number of iterations, the convergence speed was faster, and the training loss was smaller.

As shown in Table 3, the experimental results show that our proposed segment-level fusion strategy performed best when the original video was separated by eight frames. Some features in the segment will be lost due to excessive segmentation. However, too-small segment spacing will result in too many redundant features being extracted from the video sequence, and the problem of long-distance dependence before and after the long sequence will arise. Both of these will cause the performance degradation of the final network.

In order to see the impact of each module on the accuracy of the network model, we separately performed some ablation experiments for each module we proposed when the segmentation strategy interval was eight frames (see Table 4 for details). Based on the experimental results, under the same data set and training environment, our segmented feature fusion mechanism showed a 4.06% accuracy improvement on the basic network, and the segment feature fusion module only increased the simple Euclidean distance operation between vectors without more convolution operations, reducing the number of feature vectors entering the LSTM, resulting in a decrease of 0.08 GFLOPs (one billion floating-point operations per second) in the final network computation and no change in the parameter amount. After the addition of the attention mechanism, due to the addition of the convolution layer and softmax layer, the network parameter quantity increased by 12.59 M, and the calculation quantity also increased by 0.53 GFLOPs. Finally, after the addition of the attention mechanism, the network performance improved by 2.65%. Finally, our overall network performance improved by 6.69% on the basic network.

In order to further illustrate the effectiveness of our proposed method, we also compared it with several popular video motion recognition networks. The specific results are shown in Table 5. Compared with other popular algorithms, the performance of our proposed method was improved to some extent under the condition that the parameters were not different. Compared with SLOWONLY [26], the proposed method showed 17.41 GFLOPs less computation, 5.92 M more parameters and 19.46% more detection accuracy. Compared with the C3D [15] network, our parameter quantity also decreased by 39.63 M and our accuracy increased by 1.85%. Compared with SlOWFAST [26] and TSN [24], the accuracy was improved by 1.33% and 2.21%, respectively.

**Figure 9:**
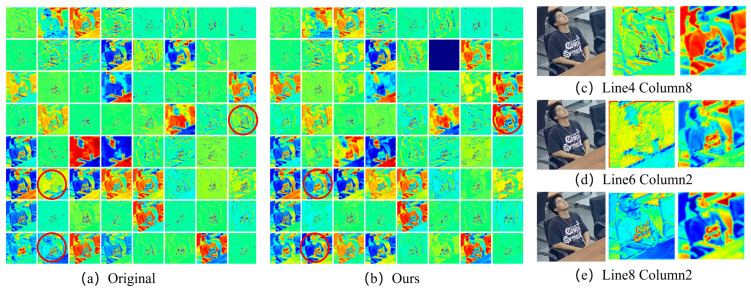
Heat map of network features before and after improvement. The subgraph (**a**) is the thermal diagram of the original network, and the subgraph (**b**) is the thermal diagram of the proposed method; subgraph (**c**–**e**) show the effect comparison between the original method and ours (red circles).

In order to further verify the effectiveness of the proposed self-attention mechanism module and segment-level feature fusion module, the fusion features in the encoding process were visualized, and the feature heat map was generated, as shown in Figure 9. Figure 9a,b are the heat maps of the network characteristics before and after the improvement, respectively, of which the details of the red circles correspond to Figure 9c–e. It can be seen that the improved feature heat map is better than the original network in terms of details, and the contour of the character feature extraction is more obvious. The experiment shows that the introduction of self-attention mechanism and fragment-level feature fusion module significantly enhanced the ability of the network model to learn long-distance dependency and attribute features, thereby improving the accuracy of target behavior difference judgment and achieving the goal of improving the overall model behavior recognition accuracy.

**Figure 10:**
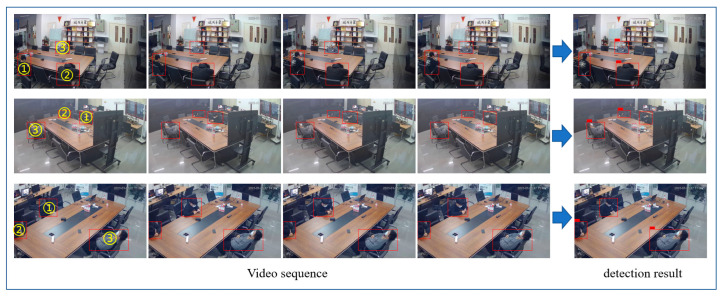
Sleep video detection results. Among them, ① ② and ③ were the target person to be tested (red squares).

In order to intuitively show the experimental results of this paper, we simulated the sleepy behavior of different postures in the laboratory, verified the video clips taken from three angles, and analyzed the sleeping behavior using the network as the detection model in this paper. Figure 10 shows the experimental results of partial video clip output. As can be seen from Figure 10, taking the first line of the video sequence as an example, target ① is in the normal sitting position using a mobile phone, target ② is in the lying down position, and target ③ is in the supine position. Drowsiness is a continuous process. In this paper, the duration of determining the drowsy state was set to 3 min. It can be seen from the graph results that both target ② and target ③ were judged to be asleep. The experiments showed that the algorithm in this paper has good generalization performance and good application value.

## 5. Conclusions

The research in this paper shows that: (1) Introducing the self-attention mechanism into the network coding layer and combining it with the ResNet50 structure to enhance the feature relationship between image sequences was effective, which helped the network fully capture fine-grained local features from the encoded image features to enhance the feature relationship between image sequences. (2) Comparing the Euclidean distance between feature vectors passing through the coding layer can be used to calculate the similarity between features and fuse the most representative and the most distinct features. This method makes the network pay more attention to the differences between image sequences, which can further improve the accuracy of sleep behavior recognition.

Compared with other behavior recognition, sleep behavior recognition is a more challenging behavioral recognition task. Currently, no effective method has been found to solve the problem of sleep behavior recognition. Because of its long behavior duration, less movement information is generated in time and space, and the problem of sleep posture may be similar to daily office representative information and space–time characteristics. Thus, the network needs to have a more sophisticated feature difference capture capability to solve the problem of sleep behavior recognition. In cognitive neuroscience, attention is an indispensable complex cognitive function of human beings. It is reflected that the human brain can intentionally or unintentionally select a small piece of useful information from a large amount of external input information to focus on processing and ignoring other information. The purpose of introducing the self-attention mechanism into the sleep recognition network is to enable the network to have the ability to self-judge the importance of features in the process of feature learning. Then, the two coded features with significant differences are combined into segment-level features instead of the original random sampling method in the segment. This effectively enhances the network’s ability to extract global features and increases the recognition accuracy of the sleep data set. The action recognition method of the long video sequence based on video segmentation feature extraction and fusion proposed in this paper has a specific reference value for action recognition under long video sequences. It can provide the technical basis for the intelligent detection of personnel sleeping behavior under security monitoring.

## Figures and Tables

**Table 1:** Corresponding accuracy of data sets with different frame intervals.

Frame Interval	Accuracy (%)
5 frames	89.20
30 frames	84.82
5 and 30 frames	85.45

**Table 2:** Experimental data distribution.

Samples	Behavior	
	Sleep	Other Sitting Behavior
Training set	1036	952
Validation set	222	204
Test set	222	204
Total samples	1480	1360

**Table 3:** Fragment-level feature segmentation and different interval experimental results.

Frame Interval	Accuracy (%)	GFLOPs	Params (M)
Four frames	93.65	64.84	38.37
Eight frames	94.71		
Sixteen frames	93.06		

**Table 4:** Ablation experiment of two modules.

Net	Accuracy (%)	GFLOPs	Params (M)
CNN-LSTM (BackBone)	88.02	64.38	25.78
+Segmentation strategy	92.08	64.30	25.78
+Attention mechanism	90.67	64.91	38.37
+Both	94.71	64.84	38.37

**Table 5:** Comparative experiments between different networks.

Net	Accuracy (%)	GFLOPs	Params (M)
SLOWONLY	75.25	82.25	32.45
C3D	92.86	-	78.00
SLOWFAST	93.38	-	34.48
TSN	92.50	-	24.74
OURS	94.71	64.84	38.37

## Data Availability

The data are not publicly available due to the privacy of sensitive information contained in the data.

## References

[1] Zeng T., Huang D. (2021). An overview of abnormal behavior detection algorithms in intelligent video surveillance systems. Comput. Meas. Control.

[2] Xie S., Zhang X., Cai J. (2019). Video crowd detection and abnormal behavior model detection based on machine learning method. Neural Comput. Appl..

[3] Shen M., Jiang X., Sun T. (2018). Anomaly detection based on Nearest Neighbor search with Locality-Sensitive B-tree. Neurocomputing.

[4] Hu X., Huang Y., Duan Q., Ci W., Dai J., Yang H. (2018). Abnormal event detection in crowded scenes using histogram of oriented contextual gradient descriptor. EURASIP J. Adv. Signal Process..

[5] Xu K., Jiang X., Sun T. (2018). Anomaly Detection Based on Stacked Sparse Coding with Intraframe Classification Strategy. IEEE Trans. Multimed..

[6] Sabokrou M., Fayyaz M., Fathy M., Moayed Z., Klette R. (2018). Deep-anomaly: Fully convolutional neural network for fast anomaly detection in crowded scenes. Comput. Vis. Image Underst..

[7] Cosar S., Donatiello G., Bogorny V., Garate C., Alvares L.O., Bremond F. (2017). Toward Abnormal Trajectory and Event Detection in Video Surveillance. IEEE Trans. Circuits Syst. Video Technol..

[8] Ye O., Deng J., Yu Z., Liu T., Dong L. (2020). Abnormal Event Detection via Feature Expectation Subgraph Calibrating Classification in Video Surveillance Scenes. IEEE Access.

[9] Mehran R., Oyama A., Shah M. Abnormal crowd behavior detection using social force model. Proceedings of the 2009 IEEE Conference on Computer Vision and Pattern Recognition.

[10] Fernando T., Denman S., Sridharan S., Fookes C. (2018). Soft + Hardwired attention: An LSTM framework for human trajectory prediction and abnormal event detection. Neural Netw..

[11] Ullah A., Muhammad K., Ser J.D., Baik S.W., Albuquerque V.H.C.d. (2019). Activity Recognition Using Temporal Optical Flow Convolutional Features and Multilayer LSTM. IEEE Trans. Ind. Electron..

[12] Martinel N., Micheloni C., Piciarelli C., Foresti G.L. (2014). Camera Selection for Adaptive Human-Computer Interface. IEEE Trans. Syst. Man Cybern. Syst..

[13] Sabokrou M., Fathy M., Hosseini M., Klette R. Real-time anomaly detection and localization in crowded scenes. Proceedings of the IEEE Conference on Computer Vision and Pattern Recognition Workshops.

[14] Wang H., Schmid C. Action Recognition with Improved Trajectories. Proceedings of the ICCV—IEEE International Conference on Computer Vision.

[15] Ji S., Xu W., Yang M., Yu K. (2013). 3D Convolutional Neural Networks for Human Action Recognition. IEEE Trans. Pattern Anal. Mach. Intell..

[16] Luo W., Liu W., Gao S. Remembering history with convolutional LSTM for anomaly detection. Proceedings of the 2017 IEEE International Conference on Multimedia and Expo (ICME).

[17] Donahue J., Hendricks L.A., Rohrbach M., Venugopalan S., Guadarrama S., Saenko K., Darrell T. (2017). Long-Term Recurrent Convolutional Networks for Visual Recognition and Description. IEEE Trans. Pattern Anal. Mach. Intell..

[18] Ullah A., Ahmad J., Muhammad K., Sajjad M., Baik S.W. (2018). Action Recognition in Video Sequences using Deep Bi-Directional LSTM With CNN Features. IEEE Access.

[19] Gammulle H., Denman S., Sridharan S., Fookes C. Two Stream LSTM: A Deep Fusion Framework for Human Action Recognition. Proceedings of the 2017 IEEE Winter Conference on Applications of Computer Vision (WACV).

[20] Li Q., Qiu Z., Yao T., Tao M., Luo J. Action Recognition by Learning Deep Multi-Granular Spatio-Temporal Video Representation. Proceedings of the 2016 ACM on International Conference on Multimedia Retrieval.

[21] Li Z., Gavrilyuk K., Gavves E., Jain M., Snoek C.G.M. (2018). VideoLSTM convolves, attends and flows for action recognition. Comput. Vis. Image Underst..

[22] Ma C.-Y., Chen M.-H., Kira Z., AlRegib G. (2019). TS-LSTM and temporal-inception: Exploiting spatiotemporal dynamics for activity recognition. Signal Process. Image Commun..

[23] Simonyan K., Zisserman A. (2014). Two-Stream Convolutional Networks for Action Recognition in Videos. Adv. Neural Inf. Process. Syst..

[24] Wang L., Xiong Y., Wang Z., Qiao Y., Lin D., Tang X., Gool L.V. (2016). Temporal Segment Networks: Towards Good Practices for Deep Action Recognition. European Conference on Computer Vision.

[25] Feichtenhofer C., Pinz A., Wildes R.P.J.I. Spatiotemporal Residual Networks for Video Action Recognition. Proceedings of the IEEE Conference on Computer Vision and Pattern Recognition (CVPR).

[26] Feichtenhofer C., Fan H., Malik J., He K. SlowFast Networks for Video Recognition. Proceedings of the 2019 IEEE/CVF International Conference on Computer Vision (ICCV).

[27] Moghadam S.M., Nevalainen P., Stevenson N.J., Vanhatalo S. (2022). Sleep State Trend (SST), a bedside measure of neonatal sleep state fluctuations based on single EEG channels. Clin. Neurophysiol..

[28] Andrillon T., Solelhac G., Bouchequet P., Romano F., LeBrun M.P., Brigham M., Chennaoui M., Léger D. (2022). Leveraging machine learning to identify the neural correlates of insomnia with and without sleep state misperception. J. Sleep Med..

[29] Zhang X., Landsness E.C., Chen W., Miao H., Tang M., Brier L.M., Culver J.P., Lee J.M., Anastasio M.A. (2022). Automated sleep state classification of wide-field calcium imaging data via multiplex visibility graphs and deep learning. J. Neurosci. Methods.

[30] Yan X., Lv W., Hua W. (2018). Statistical analysis of college students’ sleeping behavior in class based on video data. Ind. Control Comput..

[31] Shuwei Z. (2021). Research and Application of Human Behavior Recognition Algorithm for Intelligent Security Scene. Master’s Thesis.

[32] Vaswani A., Shazeer N.M., Parmar N., Uszkoreit J., Jones L., Gomez A.N., Kaiser L., Polosukhin I.J.A. (2017). Attention is All you Need. Adv. Neural Inf. Process. Syst..

[33] Tan Z., Wang M., Xie J., Chen Y., Shi X.J.A. Deep Semantic Role Labeling with Self-Attention. Proceedings of the Thirty-Second AAAI Conference on Artificial Intelligence (AAAI-18).

